# Effect of animated movie in combating child sleep health problems

**DOI:** 10.1186/s40064-015-1130-4

**Published:** 2015-07-14

**Authors:** Salim R Surani, Saherish S Surani, Sreevidya Sadasiva, Zoya Surani, Amina Khimani, Sara S Surani

**Affiliations:** Texas A&M University, 1177 West Wheeler Ave, Suite 1, Aransas Pass, TX 78336 USA; Pulmonary Associates, Corpus Christi, TX USA; University of California, Berkeley, CA USA; Harvard University, Cambridge, MA USA

**Keywords:** Sleep deprivation, Teen sleep, Video based learning, Sleep education

## Abstract

**Objective:**

Sleep deprivation among teens is a major health issue. Only 15% of teens get 8.5 h of sleep on school nights. Sleep deprivation can lead to poor grades, sleepiness and moodiness. We undertook a study to assess the prevalence of sleep habit disturbance among elementary school students in South Texas with Hispanic ethnicity predominance. We also found how much a video based on sleep education had an impact on these children.

**Method:**

Once the Corpus Christi Independent School District (CCISD) approved the collection of baseline sleep data, questionnaires were administered using the Children’s Sleep Habit Questionnaire (CSHQ.) These questionnaires were distributed prior to the viewing of the educational and animated movie KNIGHTS (Keep Nurturing and Inspiring Good Habits in Teen Sleep). Four months later, a random follow-up was performed and the children were requested to respond to the same CSHQ.

**Results:**

264 children from two elementary schools participated in this educational program. At baseline, 55.56% of the children had trouble sleeping. When the questionnaire was administered four months later, only 23.26% (p < 0.05) had trouble sleeping. Additionally, at baseline, approximately 60–70% children had some baseline bedtime resistance, anxiety dealing with sleep, issues with sleep duration and/or awakenings in the middle of the night. In the follow up questionnaire, results showed significant improvements in overall sleep habits, bedtime resistance, sleep anxiety and night awakenings amongst students (p < 0.05). However, no significant differences were seen in sleep duration and daytime sleepiness.

**Conclusion:**

Sleep deprivation and good sleep habits remain as a pervasive challenge among elementary school students. Administering an animated video about sleep education along with a provider-based education may be an effective tool for educating elementary school students and decreasing the prevalence of these sleep-related issues. Future prospective randomized studies are suggested.

## Background

Sleep deprivation and sleep problems are common among adolescents. Sleep deprivation occurs when one does not get enough sleep. It can also lead to impairment of the cognitive function (Nixon et al. 2008; Ng et al. 2009; Iwadare et al. 2013). The average adult needs about seven to 8 h of sleep per night, while adolescents need about 9 h of sleep in order to feel alert, well rested, and for optimal performance in the domain of cognitive process, school performance, mood regulation and health (Mercer et al. 1998; Anderson et al. 2009; Wolfson and Carskadon 1998).

Approximately 15 million American children are affected by inadequate sleep (Smaldone et al. 2007). Most teens do not get enough sleep as well, and only 15% of teens report sleeping at least 8.5 h on school nights (Carskadon et al. 1998). Studies have shown that teens are more inclined to have irregular sleep patterns across the week, and tend to stay up late and sleep in late on weekends. This is detrimental to their biological clock and affecting their quality of sleep. 70% of the teens have their bedtime at 10 pm or later. The most common reason accounted for delayed sleep time is attributed to homework (46%), hanging out with friends (30%), television (39%), stress (42%), or part-time job (21%), to name just a few (Noland et al. 2009).

Approximately 90% of parents feel that their children are getting adequate sleep. On the other hand 60% of adolescents reported difficulty in getting out of bed in the morning and the majority of them require their parents to wake them up for school (Carksadon et al. 2006; Wolfson et al. 2003; Crowley et al. 2006).

The study from Australian Centre for Education in Sleep (ACES) showed significant improvements of sleep knowledge were as the New Zealand trial showed significant improvements in both week and weekend sleep duration. Positive feedback regarding the program was received from both teachers and students involved in these trials. Overall, the ACES sleep education programs have proved that these programs are capable of improving sleep duration and knowledge amongst adolescents. The findings in this study show promising signs that interventions amongst adolescents can indeed improve sleep knowledge and overall sleep hygiene, thus improving sleep-related psychological, social, and health issues (Moseley and Gradisar 2009).

The ACES intervention involved implementation in a lecture-based style involving PowerPoint presentation. However, studies have shown that students prefer sleep education to be more interactive, and less monotonous. From the literature it was very clear that adolescents were encountering significant challenges due to sleep deprivation, delayed phase syndrome and challenges due to inadequate sleep. Thus, we undertook a study to assess the baseline sleep data and education of the elementary school students to help create healthy sleep habits. We designed and produced a 3D animated sleep education movie *KNIGHTS* (Keep Nurturing and Inspiring Good Habits in Teen Sleep) to help create healthy sleep habits.

## Objective

To assess the baseline sleep habit among elementary school children and to assess the role of media based interventions, in result, having a positive effect.

## Methods

For this study, permission was obtained from the CCISD (Corpus Christi Independent school District) as a part of broader sleep education program. The children underwent survey at baseline and after the intervention (animated movie). Parental consent was not required as the program was done as an educational activity, and pre and post intervention survey was conducted.

Baseline sleep data was collected from the 4th and 5th grade elementary school children by using Children Sleep Habit Questionnaire-Sleep Self Report Form (CSHQ-SSRF). Both schools have the Hispanic ethnicity predominance with 52% of their children being of Hispanic ethnicity. CSHQ-SSRF form assessed the children sleep habits in the domain of bedtime resistance, sleep onset delay, sleep anxiety, and sleep duration, night awakening and daytime sleepiness. CSHQ is a validated tool showing good internal consistency for both the community and clinical sample (Owens et al. 2000). Sleep duration, bedtime resistance, daytime sleepiness, and sleep anxiety showed the highest internal consistencies. The Sleep Self Report Form is a 26-item survey, which is designed to assess sleep domains similar to the CSHQ. The items were rated based on a 3-point scale. This ranged from usually 5–7 times a week, sometimes 2–4 times a week, and rarely 0–1 times a week. In this survey instrument, higher scores indicate more abnormal sleep (Owens et al. 2000).

Each session comprised of 45–50 min with an average of 30–40 children in each session. Trained middle school children who underwent a 2-h training session from a board certified sleep specialist and the Registered Respiratory therapist conducted the session. Training sessions included basic education about teen sleep, viewing of the movie KNIGHTS and a question and answer (Q&A) session on any sleep questions they had and questions they could have encountered during the session.

The session started with the introduction of the teen moderators, and administration of the pre-questionnaire. This was followed with the viewing of the movie KNIGHTS (approximately 19 min). This was the first 3D animated movie produced by It’s Your Life Foundation (https://www.itsyourlifefoundation.org). This can be viewed from the foundation site or from https://www.youtube.com/watch?v=RwjrXJ4e-0Y. This was followed by a Q&A session. The trained project coordinator were also available during the session to address any questions which the teen moderator may not have been able to answer.

Four months later, an unannounced random survey was carried out by the administration of the CSHQ-SSRF, again.

### Statistics

Statistical analysis was performed using R Statistical Software (Foundation for Statistical Computing in Vienna, Austria). The proportion of responses to the pre-intervention questionnaire was calculated to determine baseline sleep habits and problems. The mean scores were calculated for each question and the overall questionnaire. *T* test was performed on the mean scores (p < 0.05) to detect the differences between the pre and post-intervention. The proportion of responses of each question was compared between the pre and post-intervention groups using contingency tables with a statistical significance of (p < 0.05) which was determined using the Pearson Chi square test statistic.

## Results

The questionnaire was answered by 264 elementary school students, of which 166 were in 4th grade and 98 in 5th grade respectively. The total sample size and missing data is detailed in Table 1.

**Table 1:** Total sample and missing data

	Pre-intervention			Post-intervention		
	Grade 4	Grade 5	Both	Grade 4	Grade 5	Both
Total number of students	166	98	264	165	98	263
Missing data for at least 1 question	13	3	10	12	6	18
Response to all questions	153	95	254	153	92	245

The results are divided into 3 parts namely,

- Elementary School Pre-Intervention Data Report.
- Elementary School Post-Intervention Data Report.
- Comparison between Pre and Post-Intervention Data.

### Pre-intervention results

The Pre-intervention data is summarized in terms of mean questionnaire scores, response percentages to the questionnaire and comparison between grades 4 and 5.

**Figure 1:**
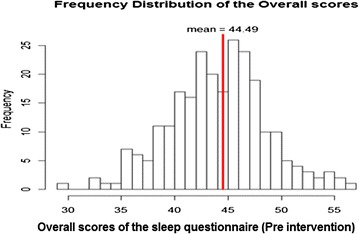
Distribution of the overall questionnaire scores for pre intervention data.

*Questionnaire scores* The sum of the scores for the whole questionnaire (questions 2 through 26) has a possible maximum score of 73 and a minimum score of 23. In the pre intervention data, the questionnaire scores ranged from 29 to 57 with a mean and median of 45, as can be seen in Figure 1.

*Response percentages* Of the 264 students, 56% had trouble sleeping and 21% did not like to sleep. Among the different blocks (Table 2), 30–50% of students had major sleep issues in block 1 of the questionnaire (bedtime resistance). In the block 2, 3, 4, 5 and 6, 10–30% of students had problems. 50% of the students rarely slept in the same bed and were not ready to sleep at the usual bedtime. Only a third of the students fell asleep within 20 min. 36% rarely felt rested after a good nights sleep (Table 3).

**Table 2:** Sleep questionnaire with answers are coded as (1), (2), and (3)

	Questionnaire	Answers		
1	Who in your family sets the rules about when you got to bed?			
2	Do you have trouble sleeping	Yes (2)	No (1)	
3	Do you like to sleep	Yes (1)	No (2)	
	Block 1—bedtime resistance			
4	Do you go to bed at same time every night on school nights?	Usually (1)	Sometimes (2)	Rarely (3)
5	Do you fall asleep in the same bed every night?	Usually (1)	Sometimes (2)	Rarely (3)
6	Do you fall asleep alone?	Usually (1)	Sometimes (2)	Rarely (3)
7	Do you fall asleep in parents’, brother’s or sister’s bed?	Usually (3)	Sometimes (2)	Rarely (1)
9	Do you fight with your parents about going to bed?	Usually (3)	Sometimes (2)	Rarely (1)
10	Is it hard for you to go to bed?	Usually (3)	Sometimes (2)	Rarely (1)
11	Are you ready for bed at your usual bedtime?	Usually (1)	Sometimes (2)	Rarely (3)
15	Do you stay up late when your parents think you are asleep?	Usually (3)	Sometimes (2)	Rarely (1)
	Block 2—sleep onset delay			
8	Do you fall asleep in about 20 minutes?	Usually (1)	Sometimes (2)	Rarely (3)
	Block 3—sleep anxiety			
12	Do you have a special thing you bring to bed?	Usually (3)	Sometimes (2)	Rarely (1)
13	Are you afraid of the dark?	Usually (3)	Sometimes (2)	Rarely (1)
14	Are you afraid of sleeping alone?	Usually (3)	Sometimes (2)	Rarely (1)
	Block 4—sleep duration			
16	Do you think you sleep too little?	Usually (3)	Sometimes (2)	Rarely (1)
17	Do you think you sleep too much?	Usually (3)	Sometimes (2)	Rarely (1)
	Block 5—night waking’s			
18	Do you wake up at night when parents think you are asleep?	Usually (3)	Sometimes (2)	Rarely (1)
19	Do you have trouble falling back to sleep if you wake up during the night?	Usually (3)	Sometimes (2)	Rarely (1)
20	Do you have nightmares?	Usually (3)	Sometimes (2)	Rarely (1)
21	Does pain wake you up at night? Where is that pain?	Usually (3)	Sometimes (2)	Rarely (1)
22	Do you sometimes go to someone else bed during the night? If yes who?	Usually (3)	Sometimes (2)	Rarely (1)
	Block 6—daytime sleepiness			
23	Do you have trouble waking up in the morning?	Usually (3)	Sometimes (2)	Rarely (1)
24	Do you feel sleepy during the day?	Usually (3)	Sometimes (2)	Rarely (1)
25	Do you take naps during the day?	Usually (3)	Sometimes (2)	Rarely (1)
26	Do you feel rested after a night’s sleep?	Usually (1)	Sometimes (2)	Rarely (3)

**Table 3:** Response percentages to the questionnaire (pre intervention data)

Response percentages to the questionnaire (pre intervention data, both grades combined)				
Quest#		Yes (%)	No (%)	
2	Trouble sleeping	55.56	44.44	
3	Likes to sleep	78.79	21.21	

		Rarely (%)	Sometimes (%)	Usually (%)
	Bedtime resistance			
4	Goes to bed at same time	38.64	32.20	29.17
5	Falls asleep in same bed	50.95	28.14	20.91
6	Sleeps alone	47.73	27.27	25.00
7	Falls asleep in other’s bed	41.06	30.80	28.14
9	Fights with parents to go to bed	58.71	31.06	10.23
10	Hard to go to bed	39.69	32.44	27.86
11	Ready to sleep at usual bedtime	51.52	25.00	23.48
15	Stay up late when parents think you are asleep	41.29	28.03	30.68
	Sleep duration			
8	Falls asleep in 20 min	31.44	35.61	32.95
	Sleep anxiety			
12	Take a special thing to bed	44.70	33.33	21.97
13	Afraid of dark	51.15	25.19	23.66
14	Afraid of sleeping alone	46.59	35.98	17.42
	Sleep duration			
16	Sleeps too little	49.43	31.56	19.01
17	Sleeps too much	53.79	30.68	15.53
	Night awakening			
18	Wakes up at night when parents think you are asleep	56.82	29.17	14.02
19	Trouble falling back asleep after waking up	51.89	31.06	17.05
20	Have nightmares	48.11	33.71	18.18
21	Wakes up due to pain	53.08	33.08	13.85
22	Goes to other’s bed during night	53.23	35.74	11.03
	Daytime sleepiness			
23	Trouble waking up in morning	44.70	34.85	20.45
24	Feels sleepy during day	48.11	39.77	12.12
25	Take naps during day	55.30	29.55	15.15
26	Feels rested after a night’s sleep	35.74	31.94	32.32

Comparison between grades 4 and 5: We also did the comparison between the students between grade 4th and 5th grade. There is a significant difference in the percent of students who had trouble sleeping, 63% in grade 4 compared to 43% in grade 5. (Chi square p value = 0.0), as seen in Table 4.

**Table 4:** Comparing pre-intervention data between Grade 4 and 5

Comparing pre-intervention data between Grade 4 and 5							
Quest #		Grade 4		Grade 5		t test	chisq
		Yes (%)	No (%)	Yes (%)	No (%)		
2	Trouble sleeping	63.80	36.20	42.86	57.14	–	0.00*
3	Likes to sleep	79.52	20.48	77.55	22.45	–	0.82

		Rarely (%)	Usually (%)	Rarely (%)	Usually (%)	t test	chisq
10	Hard to go to bed	45.18	22.89	30.21	36.46	0.01*	0.02*
11	Ready to sleep at usual bedtime	48.80	23.49	56.12	23.47	0.53	0.00*
15	Stay up late when parents think you are asleep	37.35	34.34	47.96	24.49	0.05*	0.16
16	Sleeps too little	54.55	16.36	40.82	23.47	0.04*	0.09
18	Wakes up at night when parents think you are asleep	53.61	17.47	62.24	8.16	0.05*	0.1
24	Feels sleepy during day	52.41	9.04	40.82	17.35	0.03*	0.07
25	Takes naps during day	59.04	9.64	48.98	24.49	0.01*	0.01*
26	Feels rested after a night’s sleep	30.30	39.39	44.90	20.41	0.00*	0.00*

* Statistically significant (p < 0.05).

The responses to the questions whether it’s hard to go to bed, whether they take naps during the day and feel rested after a night’s sleep, are significantly different between the 4th and 5th grades using the t-test and the Chi square test. Additionally, t-test showed a significant difference between the grades for the questions of staying up late when parents think they are asleep, whether they sleep too little, if they wake up at night and if they feel sleepy during the day. The summaries of the questions having significant p-values are shown in Table 4.

### Post-intervention results

The Post intervention data is summarized in terms of mean questionnaire scores, comparison between pre and post interventions in terms of proportion of responses to the questionnaire, Chi square and t-tests.

**Figure 2:**
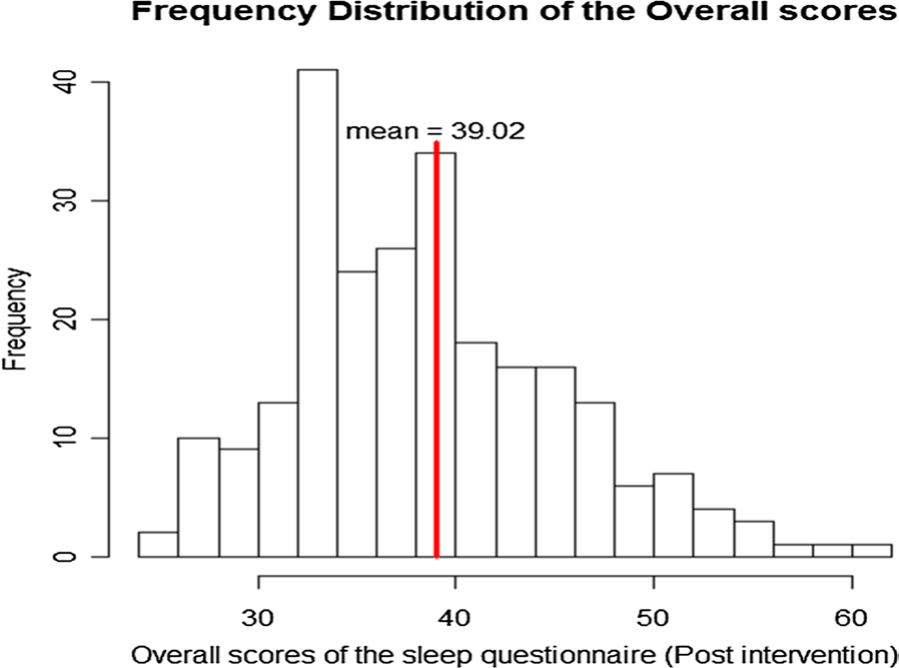
Distribution of the overall questionnaire scores for post intervention data.

The overall questionnaire scores have a mean of 39 and median of 38, which has decreased compared to the pre-intervention mean and median score of 45 (Figure 2).

For most of the questions, responses have improved by the post-intervention. Among the different blocks, block 1 (Bedtime resistance) has the biggest change in the percentages of responses. Students who usually slept in the same bed increased from 21 to 76%. Students who were not ready to sleep at the usual bedtime are 26%, which decreased from 52% previously. Those who stayed up late decreased from 31 to 13%. It increased to 56% of students whom usually felt rested after a good night’s sleep, which was previously 32%. The percentages of responses are detailed in Table 5.

**Table 5:** Response Percentages comparing pre-intervention and post intervention data

Quest #	Response percentages to the questionnaire	Pre intervention		Post intervention	
		Rarely (%)	Usually (%)	Rarely (%)	Usually (%)
	Bedtime resistance				
4	Goes to bed at same time	38.64	29.17	20.15*	38.40*
5	Falls asleep in same bed	50.95	20.91	5.72*	75.95*
6	Sleeps alone	47.73	25.00	14.88*	67.94*
7	Falls asleep in other’s bed	41.06	28.14	73.18*	13.79*
9	Fights with parents to go to bed	58.71	10.23	62.74	12.93
10	Hard to go to bed	39.69	27.86	53.82*	15.23*
11	Ready to sleep at usual bedtime	51.52	23.48	25.67*	34.87*
15	Stay up late when parents think you are asleep	41.29	30.68	57.41*	12.55*
	Sleep onset delay				
8	Falls asleep in 20 min	31.44	32.95	29.39*	29.01
	Sleep anxiety				
12	Takes a special thing to bed	44.70	21.97	50.38	30.92
13	Afraid of dark	51.15	23.66	70.34*	12.93*
14	Afraid of sleeping alone	46.59	17.42	76.05*	5.70*
	Sleep Duration				
16	Sleeps too little	49.43	19.01	49.43	12.55*
17	Sleeps too much	53.79	15.53	56.65*	9.88*
	Night awakening				
18	Wakes up at night when parents think you are asleep	56.82	14.02	54.75	12.93*
19	Trouble falling back asleep after waking up	51.89	17.05	43.89	19.47
20	Have nightmares	48.11	18.18	64.89	8.40*
21	Wakes up due to pain	53.08	13.85	68.46	7.69*
22	Goes to other’s bed during night	53.23	11.03	71.37	6.49*
	Daytime sleepiness				
23	Trouble waking up in morning	44.70	20.45	35.50	24.05
24	Feels sleepy during day	48.11	12.12	38.55	14.89
25	Takes naps during day	55.30	15.15%	61.83	12.21*
26	Feels rested after a night’s sleep	35.74	32.32	16.41*	55.73

* Denotes an increase in the percentage of students who have responded positively post intervention.

There is a significant decrease in the percent of students who had trouble sleeping, from 56 to 23% after intervention (Chi square p value = 0.0) as seen in Table 6. Out of the 25 questions, Chi square tests showed statistically significant difference between the pre and post-intervention data for 15 questions. For most questions in Block 1, Block 3 and Block 5, there is a significant difference between the pre and post-intervention as shown by Chi square tests (Table 7). The t-tests showed similar results to the Chi square tests. The overall questionnaires mean scores are statistically significant between the pre and post groups (p value = 0). T tests showed that mean scores for Block 1: Bedtime Resistance (p value = 0), Block 3: Sleep anxiety (p value = 0) and Block 5 : Night awakenings (p value −0), are all statistically significant between pre and post-intervention data. The mean scores in Block 2, Block 4 and Block 6, Daytime Sleepiness and Sleep Duration were not significantly different (Table 7).

**Table 6:** Comparing pre-intervention and post intervention data (questions 2 and 3)

Quest #		Pre intervention		Post intervention		Chisq (p value)
		Yes (%)	No (%)	Yes (%)	No (%)	
2	Trouble sleeping	56	44	23	77	0.00*
3	Likes to sleep	79	21	77	23	0.82

* Denotes significant p values at 0.05 significance level

**Table 7:** Comparing pre-intervention and post intervention data (Grades 4 and 5 combined)

Comparing pre-intervention and post intervention data			
		t test	chisq
	Questions 2 to 26 (overall score)	0.00*	NA**
	Block 1—Bedtime Resistance	0.00*	NA
4	Do you go to bed at same time every night on school nights?	0.00*	0.00*
5	Do you fall asleep in same bed every night?	0.00*	0.00*
6	Do you fall asleep alone?	0.00*	0.00*
7	Do you falls asleep in parents’, brother’s or sister’s bed?	0.00*	0.00*
9	Do you fight with your parents about going to bed?	0.81	0.19
10	Is it hard for you to go to bed?	0.00*	0.00*
11	Are you ready for bed at your usual bedtime?	0.00*	0.00*
15	Do you stay up late when parents think you are asleep?	0.00*	0.00*
	Block 2—sleep onset delay		
8	Do you fall asleep in about 20 min?	0.72	0.36
	Block 3—sleep anxiety	0.00*	NA
12	Do you have a special thing you bring to bed?	0.68	0.00*
13	Are you afraid of the dark?	0.00*	0.00*
14	Are you afraid of sleeping alone?	0.00*	0.00*
	Block 4—sleep duration	0.10	NA
16	Do you think you sleep too little?	0.28	0.08
17	Do you think you sleep too much?	0.20	0.15
	Block 5—night awakening	0.00*	NA
18	Do you wake up at night when parents think you are asleep?	0.86	0.73
19	Do you have trouble falling back to sleep if you wake up during the night?	0.12	0.18
20	Do you have nightmares?	0.00*	0.00*
21	Does pain wake you up at night? Where is that pain?	0.00*	0.00*
22	Do you sometimes go to someone’s bed during the night? If yes who?	0.00*	0.00*
	Block 6—daytime sleepiness	0.07	NA
23	Do you have trouble waking up in the morning?	0.07	0.10
24	Do you feel sleepy during the day?	0.06	0.09
25	Do you take naps during the day?	0.13	0.30
26	Do you feel rested after a night’s sleep?	0.00*	0.00*

* Denotes significant p-values at 0.05 significance level.

** *NA* not applicable.

## Discussion

Our study aimed at assessing baseline sleep habits and behaviors in elementary school children. Baseline data indicated a significant percentage of students have sleep problems. These findings are consistent with the previous studies done on elementary school children (Owens et al. 2000; Mindell et al. 2009; Blader et al. 1997).

### Baseline prevalence of sleep problems

In the present study, a high percent of students experienced problems in the areas of bedtime resistance, sleep onset delay, sleep anxiety, night awakenings and daytime sleepiness. Proportions of students affected by certain sleep problems are very similar to previous studies while other sleep problems affect a higher percentage of students.

The highest percentages of challenges were found in the Bedtime Resistance Category. 41% showed resistance with parents to go to bed at least 2 nights a week, with 10% showing resistance 5–7 nights a week. This result is in line with other studies. A single bed time resistance item on the CSHQ, 37.6% of the sample reported this behavior at least two times a week (Owens et al. 2000). In another study 27% children showed bedtime resistance (Blader et al. 1997). National Sleep Foundation (NSF) poll reported that 42% children stall to go to bed (Mindell et al. 2009). 20% of children in our study had trouble waking up 5–7 days/week. NSF poll reported 29% had difficulty waking in the morning at least a few days or nights a week (Mindell et al. 2009) and 17% had morning wake up problems according to (Blader et al. 1997).

Our study showed higher percentages of sleep-onset delays, whereas 31% rarely slept within 20 min. NSF poll reported 22% take more than 20 min to fall asleep (Mindell et al. 2009) whereas 11.3% had sleep-onset delays. Similarly we found 43% reported night wakening at least 2 times a week and 14% wakening at least 5 times a week, which is higher than 27% night wakening at least 2 times a week, for the single item question in CSHQ (16) and 14% children waking at least one time per night (Mindell et al. 2009). We found that 55% take a nap rarely whereas NSF poll reported 94% took no nap at all (Mindell et al. 2009).

### Effect of intervention on sleep patterns and habits

The study compared sleep patterns and habits through the pre and post interventions. We noticed an improvement in the sleep behaviors especially in the areas of bedtime resistance, sleep anxiety and night awakening after the *KNIGHTS* program. There is a significant decrease in the percent of students who had trouble sleeping, from 56 to 23% after the intervention, suggesting the effectiveness of video base and Q&A programs.

Studies have reported that sleep education resulted in improved sleep behavior (Rossi et al. 2002) and increased sleep duration (Rossi et al. 2002; Blunden et al. 2012; Vo et al. 2003; Bakotic et al. 2009). Previous studies that measured changes in sleep parameters (e.g., sleep hygiene practices and sleep duration), showed mixed results. Some studies showed improvement with sleep hygiene whereas another study reported earlier bed and wake times on both school and weekend nights for students receiving sleep education (Vo et al. 2003).

School based sleep programs seek to improve sleep knowledge using different education programs assuming that will improve sleep habits. Many studies have showed that sleep knowledge significantly increased with school based sleep education program (Moseley and Gradisar 2009; Blunden et al. 2012; Bakotic et al. 2009; De Sousa IC et al. 2007; Cortesi et al. 2004; Cain et al. 2011; Azevedo et al 2008; Cassoff et al. 2013). However, school-based sleep promotion programs usually do not succeed in improving and maintaining sleep behavioral changes (Blunden et al. 2012; Cassoff et al. 2013). Sleep parameters such as sleep patterns, sleep quality and sleep habits did not change significantly after the sleep programs (Moseley and Gradisar 2009; Vo et al. 2003; De Sousa IC et al. 2007; Cain et al. 2011). In addition to sleep education, some programs have used cognitive and behavioral strategies, but they have not been very successful in improving sleep behavior (Moseley and Gradisar 2009).

Studies have reported the benefits of school-based programs to educate and improve behaviors in adolescents for many health related issues such as diet, physical activity, eating disorders, drug abuse and depression (Wade et al. 2003; Orlando et al. 2005; Possel et al. 2005; Kitzman-Ulrich et al. 2010; Harris et al. 2009; Hatzis and Kafatos 2010).

Interactive programs with student participation are more effective than brief lecture-style programs in changing behavior (Clarke et al. 1993; Stice and Shaw 2004). No standardized assessment measures exist for testing sleep knowledge in children. Hence studies construct and develop their own questionnaires. Blunden et al. therefore highlight the need of use for a standard sleep knowledge measure (Blunden et al. 2012).

## Limitations and future directions

Our study has several limitations. The study sample had a Hispanic ethnicity predominance of 52% children being of Hispanic ethnicity. However no data was collected on the race/ethnicity of individual students. We also did not collect any information regarding the gender of the students. Hence the responses were not classified according to ethnicity and gender.

Ethnicity and gender have significant effects on sleep behaviors. Non-Caucasian children reported later bedtimes and had shorter sleep duration than Caucasian children (Biggs et al. 2013; Gulliford et al. 1990; Liu et al. 2005; Spilsbury et al. 2004). Previous studies have reported that boys had shorter sleep duration than girls on non-school nights (Biggs et al. 2013; Gulliford et al. 1990; Liu et al. 2005; Ng et al. 2005). Moore et al. (2011) found that gender and minority ethnicity was significantly associated with sleep duration, with girls and non-minority adolescents obtaining more sleep. Another sleep study conducted on minority children found that gender had significant effect on sleep duration, where girls aged 11–12 year slept 0.3 h/days less than boys (Wong et al. 2013). Presence of TVs in the child’s bedroom contributes to sleep problems (Owens et al. 1999) and a higher percent of Hispanic (74%) children compared with white children (22%) have TVs in their bedroom (Taveras et al. 2009). Sleep education with educational leaflets was more effective in female adolescents compared to male adolescents (Bakotic and Koscec 2009). Identifying the gender and racial makeup of the sample will give more insight into the sleep problems and effectiveness of treatments.

In previous studies using CSHQ, the questionnaire was answered by students, teachers and/or parents (Iwadare et al. 2013; Owens et al. 2000). Their responses were correlated to get a better picture of the sleep behaviors. It has also been shown that parents can influence children’s sleep habits (Brand et al. 2009). Our study has only the student’s answer the questionnaire. In future studies, we will include parents and teachers to take the questionnaire as well, which will help us to objectively evaluate sleep habits and behavior.

Students were retested with the questionnaire 4 months after the intervention and the results showed significant results. This is evidence that our *KNIGHTS* program is successful. However, all the students who answered the pre-intervention questionnaire underwent the intervention and there was no control class or students unlike some previous studies, which have used controls (Brown et al. 2006) and randomized controlled design (Moseley and Gradisar 2009; Rossi et al 2002; Vo et al. 2003; Bakotic et al. 2009; Cortesi et al. 2004). Therefore we cannot completely exclude other factors influencing this improved sleep behavior. Future studies are suggested to look into these issues.

## Conclusion

There are significant sleep issues among elementary school children. The *KNIGHTS* sleep education program has been beneficial. The study demonstrated an increase in positive sleep behaviors and decrease in sleep problems. Our study is not without limitations. Future studies can include gender and racial make-up of the sample, involving parents and teachers in the program, using controls and the study, a randomized controlled study. Beneficial effect of the video based learning provides another venue for the educator to look into, to improve the sleep habits and behavior.
